# Nutritional status, exclusive breastfeeding and management of acute respiratory illness and diarrhea in the first 6 months of life in infants from two regions of Indonesia

**DOI:** 10.1186/s12887-017-0966-x

**Published:** 2017-12-21

**Authors:** V. Oktaria, K. J. Lee, J. E. Bines, E. Watts, C. D. Satria, J. Atthobari, H. Nirwati, C. D. Kirkwood, Y. Soenarto, M. H. Danchin

**Affiliations:** 1 Pediatrics Research Office, Child Health Department, Faculty of Medicine, Universitas Gadjah Mada/ DR Sardjito Hospital, Yogyakarta, Indonesia; 2 Department of Pediatrics, Faculty of Medicine, Dentistry and Health Sciences, The University of Melbourne, Melbourne, Australia; 3 Murdoch Childrens Research Institute (MCRI), The Royal Children Hospital, Melbourne, Australia; 4 Department of Pharmacology & Therapy, Faculty of Medicine, Universitas Gadjah Mada, Yogyakarta, Indonesia; 5 Department of Microbiology, Faculty of Medicine, Universitas Gadjah Mada, Yogyakarta, Indonesia; 6 Bill and Melinda Gates Foundation, Seattle, USA

**Keywords:** Nutritional status, Case-management, Indonesian infants, Breastfeeding, Acute respiratory illness

## Abstract

**Background:**

Infant morbidity and mortality rates remain high in Indonesia, with acute respiratory illnesses (ARI) and diarrhea the leading two health problems in children under 5 years. We aimed to describe the nutritional status, feeding practice and case management of ARI and diarrhea of infants from two regions of Indonesia during the first 6 months of life.

**Methods:**

This study was an observational study conducted in parallel to an immunogenicity and efficacy trial of an oral rotavirus vaccine (RV3-BB) in the Klaten and Yogyakarta regions, Indonesia. Mothers were interviewed at 3 time points: within the first 6 days of their infant’s life, and at 8–10 and 22–24 weeks of age. Questions asked included pregnancy history, infant nutritional status, feeding status and health of infants within up to 2 weeks prior to the assessment.

**Results:**

Between February 2013 and January 2014, 233 mother-infant pairs were recruited. 60% (136/223) of infants were exclusively breastfed (EBF) until 6 months of age with the strongest support for EBF reported by mothers themselves 70% (101/223) and 25% (36/223) from their partners. At 6 months, 6% (14/223) of infants were underweight and severely underweight; 4% (8/ 223) wasted and severely wasted; and 12% (28/223) were stunted and severely stunted. Non-recommended medication use was high, with 54% (21/39) of infants with reported cough within 2 weeks of an assessment receiving cough medication, 70% (27 /39) an antihistamine, 26% (10/39) a mucolytic and 15% (6 /39) an oral bronchodilator. At age 22–24 week, infants with reported diarrhea within 2 weeks of an assessment had low use of oral rehydration solutions (ORS) (3/21;14%) and zinc therapy (2/ 21;10%).

**Conclusion:**

In this unique observational study, breastfeeding rates of 60% at 6 months were below the Indonesian national target of >75%. Adherence to WHO guidelines for management of ARI and diarrhea was poor, with high use of non-recommended cough medications and oral bronchodilators in the first 6 months of life and low use of ORS and zinc therapy. Ongoing education of primary health care workers and parents regarding management of common illness is needed in Indonesia.

## Background

Despite the improvement in the health status of Indonesian infants in the last two decades, mortality rates remain high, estimated at 24.5 deaths per 1000 live births in 2013 [1, 2]. Acute respiratory illnesses (ARI) and diarrhea are the leading causes of mortality in Indonesian children under 5 years of age [3] and were the main targets of the fourth millennium development goal (MDG) to reduce child mortality by two thirds by 2015 [3]. Globally, Indonesia has amongst the highest incidence of ARI, with approximately 6 million new ARI cases per year in children under 5 years estimated in 2008 [4]. In 2013, the prevalence of ARI and diarrhea was 0.24 and 3.5% respectively as reported in the 2013 Indonesia Basic Health Research (IBHS), [6] a national cross-sectional survey to capture the health problems in 33 provinces in Indonesia (*n* = 1,027,763) such as any episodes of ARI and diarrhea in the 4 and 2 weeks prior to the survey and case management of diarrhea with Oral Rehydration Solution (ORS) and zinc. The IBHS also reported that malnutrition was common, with 6.8% of children under 5 years of age defined as wasted, 5.3% as severely wasted, 19.2% as stunted and 18% as severely stunted in 2013 [5, 6]. Importantly, macronutrient deficiencies have been associated with an increased risk of developing ARI and diarrhea, [7] with a 4-fold increase in ARI-related deaths in severely malnourished children compared to children with normal nutritional status [8, 9].

Identifying effective disease prevention and management strategies in Indonesia remains an important goal to decrease the morbidity and mortality rate from ARI and diarrhea. The World Health Organization (WHO) and The United Nations Children’s Emergency Fund (UNICEF) recommend exclusive breastfeeding (EBF) for 6 months, continued breastfeeding up to 2 years of age and improvement of case management in health facilities for protection, prevention and treatment of pneumonia and diarrhea [11]. Similarly, the Indonesian government recommends EBF for the first 6 months to improve nutritional status and to provide additional protection against ARI and diarrhea [7–9, 12–15] but the national coverage of EBF was reported by the Ministry of Health to be only 54% at 6 months in 2013 [5]. Furthermore, the routine national immunisation program covers BCG, hepatitis B, polio, DPT, HiB and measles vaccines, with with rotavirus and pneumococcal vaccines currently limited to the private sector [10].

One of challenges of case management in health facilities in developing countries is poor adherence to available guidelines to manage ARI and diarrhea [16, 17], resulting in inaccurate assessment of the signs and symptoms which contribute to incorrect diagnosis, inappropriate treatment [16–18] and poor outcomes [19, 20]. In Indonesia, the current available guidelines for ARI case management from the Indonesian Pediatric society and WHO are symptomatic relief and maintenance of adequate oral hydration for the treatment of Upper Respiratory Tract Infections (URTI) and antibiotics for pneumonia [21, 22]. However, current case management practice, such as the prescription of medications and how the WHO and local pediatric recommendations were followed for ARI and diarrhea in South East Asia, including Indonesia, is not well described.

We aimed to describe the nutritional status, feeding practice and case management of ARI and diarrhea of infants enrolled in a rotavirus vaccine clinical trial during the first 6 months of life in the Klaten and Jogjakarta regions in Indonesia. In particular this study focuses on the rate of EBF, the infants’ nutritional status assessed at three time-point visits and the medication prescribed for ARI and diarrhea. We hypothesized that [1] poor nutritional status would be common in infants, [2] rates of EBF would be high and that [3] the management of acute ARI and diarrhea would not be consistent with the available guidelines in Indonesia.

## Methods

We conducted an observational study, in parallel to a clinical trial of an oral rotavirus vaccine (RV3-BB) in the Klaten and Yogyakarta regions, Indonesia.

### Study location and population

This study was conducted at the 11 study sites involved in the Phase IIb RV3-BB Rotavirus Vaccine Clinical Trial from 14th February 2013 and 8th January 2014, including nine Primary Health Care Centers and two hospitals, in Klaten (rural, Central Java province) and Sleman (semi-urban, Yogyakarta province). Selection of these Primary Health Care Centers was to provide information from rural and semi-urban villages. Both the Klaten and Sleman regions are located in the Java, the most densely populated island in Indonesia. The Klaten region is situated in central java, on the border between the central java and Yogyakarta provinces. The region is divided into 26 districts with a population of just over 1.2 million people [23]. Sleman, is situated in Yogyakarta province and is more urbanized, with a population of 1,167,481 people in 2015 [24]. In 2010, the proportion of famillies living in poverty in Klaten was slightly higher compared to Sleman, (17.5% vs. 10.7%) with the national level at 13.3% [25–27].

### Recruitment to this study

This study recruited participants from those who had given consent to participate in a Phase IIb Vaccine Clinical Trial of the RV3-BB. The main vaccine trial randomised 1649 participants to receive either vaccine or placebo, with follow-up to 18 months of age to assess vaccine efficacy. To fulfill one of the trial’s secondary objectives immune response was assessed in a predetermined subgroup of 282 of the trial participants. The Phase IIb vaccine trial is trial was registered within the Australian New Zealand Clinical Trial Registry (https://www.anzctr.org.au, trial registration ID ACTRN12612001282875).

After delivery, potential trial participants attended either the hospital or Primary Health Care Center at 0–5 days of age for trial eligibility assessment prior to the administration of the first dose of investigational product. The main inclusion criteria for the trial were full term infants in good health with a birth weight between 2.5-4 kg inclusive. The main exclusion criteria included any medical, psychiatric, or social conditions of a parent/guardian that in the opinion of the investigator would prevent the neonate’s parent(s)/guardian(s) from giving proper informed consent or from complying with the study protocol; neonates with known or suspected suppressed immune systems, bleeding diathesis, or those who had received blood products or other investigational products; neonates with an HIV positive mother; infants in whom the Expanded Program on Immunization vaccines were contraindicated; and moderate or severe illness 48 h preceding randomisation. Once participants were deemed eligible to enter the immunogenicity study, mothers were invited to participate in and provide consent for our study. The study involved two components:

1. Face-to-face interviews to collect information on nutritional status, feeding practice and case management of ARI and diarrhea; as well as.
2. Collection of breast-milk samples for analysis of the correlation of maternal antibodies with vaccine take (analyses ongoing; data not presented).

Our study commenced a month after 49 participants had already been recruited in the immunogenicity study. Of the remaining 233 participants, we invited each mother to provide consent for our study. We approached the remaining 233 trial participants. In total 233 out of 282 mothers in the immunogenicity study of the trial were enrolled in this study.

### Data collection

#### Case report forms (CRFs)

Separate study specific CRFs were designed to capture the required data at each of the three follow-up assessment time-points in this study through face-to-face interview with the mother. The time-points for the three visits (0–5 days, 8–10 and 22–24 weeks of age) were in alignment with the visits for the trial’s investigational product (IP) dosing, which occurred at the hospitals or primary health care centers.

Information on the antenatal period, including the mother’s background and weight gain during pregnancy, monthly family income, living environment, health and pregnancy as well as the baby’s health status after delivery were collected at the initial interview. The second and third interviews focused on the baby’s nutrition, feeding status and health, including medications prescribed for ARI or diarrhea, as recalled by the parents within the preceding 2 weeks. Infant’s weight and length were recorded during visits at the vaccination clinic or sought from medical records when face-to face visits were unsuccessful within 2 weeks window time of scheduled meeting. Additional data were obtained from medical records held at the local Primary health care clinics (PHCs) when any illness necessitating a clinical visit within the preceding 2 weeks was reported by mothers. Phone interviews were performed when face-to-face interviews at clinics were not possible and home visits were attempted if parent(s) were un-contactable by phone.

#### Clinical definitions

Socio economic status was captured using monthly family income and defined as low if income was < IDR 1,000,000 (< USD 75), medium for income between IDR 1,000,000 to IDR 5,000,000 (USD 75–375) and high for income > IDR 5,000,000 (more than USD 375) per month. EBF was defined as infants who were exclusively breast fed at the time of the assessment with no additional food or formula. Strongest support of EBF was defined as who was reported by the mother to provide the strongest support to continue EBF and the option was herself, the father or any relative or friend. Mothers were asked for reasons if they had stopped breastfeeding before the assessment.

Infant nutritional status was determined by height and weight and was classified according to WHO definitions, where <2 standard deviations (SD) below the mean for weight for age (underweight), low height/length for age (stunted), and low weight for height/length (wasted), were classified as moderate acute malnutrition - MAM; and <3 SDs below the mean for low weight for age (severely underweight), low height/length for age (severely stunted), and low weight for height/length (severely wasted) were classified as severe acute malnutrition (SAM). Weight for length > 1 SD and ≤2 SD above the mean was classified as possible risk of overweight, > 2 SD and ≤3 SD above the mean was classified as overweight and weight for length > 3 SD above the mean was classified as obese. Any respiratory symptom, such as runny nose and wheezing, associated with cough with or without fever was defined as “ARI with cough”. “ARI without cough” was defined as any respiratory symptom as above without cough, with or without the presence of fever. Diarrhea was defined as three or more stools in a 24-h period that were looser than normal.

#### Ethics

Ethics approval for the Phase IIb rotavirus vaccine trial and this study was obtained from the Faculty of Medicine, University of Gadjah Mada in Indonesia (KE/ FK/ 465/EC and KE/FK/788/EC) and Royal Children Hospital, Melbourne, Australia (HREC No. 32060 and 34212).

#### Data analysis

The demographic characteristics, environmental factors, health of the pregnancy and delivery details of the study population are presented as means ± SDs or medians and interquartile ranges (IQR) for non-normal data (continuous variables), and numbers and proportions for categorical variables. Nutrition, feeding status and health of the infant are presented at the 3 time-points (0–6 days after birth, 8–10 weeks and 22–24 weeks of age). The EBF prevalence as well as management of ARI and diarrhea in those who report an episode of ARI/diarrhea in the preceding 2 weeks are presented as proportions. STATA version 11.0 was used to perform all data analyses.

## Results

In total, 233 out of 282 (83%) participants in the trial’s immunogenicity sub study were enrolled in this study between February 2013 and January 2014. The majority of participants were from rural Klaten (188/233; 81%) and semi-urban Sleman (45/233;19%).

### Maternal demographics

The median (IQR) for maternal age was 29 (range: 24–34) years, with the majority of mothers from rural regions (81%) (Table 1). The majority of mothers were predominantly unemployed (157/233; 67%), of low socio-economic status (188/233; 81%), with high school as the highest education level attained. Regular daily intake of any iron supplements during pregnancy was high (226/233; 97%). Calcium supplementation was also high (171/233; 77%), with only 57% (126/233) of mothers drinking fortified milk (calcium, iron, folic acid and vitamins) and only 45% (99/223) took vitamin C tablets during pregnancy. Most mothers had a normal delivery (186/233; 80%), with few caesarean deliveries (33/233; 14%) and a low reported complication rate (13/233; 6%).

**Table 1:** Demographic Characteristics of parents at the time of delivery

	Total *N* = 233
Mothers age (years), median (IQR)	29 (24–34)
Mother’s highest education, N (%)	
Middle school or less	82 (35)
High school	128 (55)
University	22 (9)
Other	1 (0)
Mother’s Occupation, N (%)	
Unemployed	157 (67)
Part-time employment	15 (6)
Casual employment	19 (8)
Full-time employment	42 (18)
Family Income per month, N (%)	
Low (< 75 USD)	188 (81)
Middle (75–375 USD)	36 (16)
High (> 375 USD	1 (0)

### Infant’s nutritional status

The majority of infants had a normal weight for age and length for age at all three time points. However, despite all babies having a birth weight of >2.5 kg at birth, at 8–10 and 22–24 weeks of age, 5% of infants had weight for age and weight for length for age < − 2SD (Table 2). At birth, 8–10 and 22–24 weeks of age, 5% (11/233), 5% (11/228), and 9% (21/223), of infants were stunted and 2% (4/233), 3% (6/228) and 3% (7/223), of infants were severely stunted respectively. The proportions that were overweight and obese at birth, at 8–10 and 22–24 weeks of age were less than 5% but the proportion of infants who were at risk of overweight (weight for length > 1 SD and ≤2 SD) was 20% (44/233), 18% (41/228), and 16% (37/223), respectively. The majority of infants with normal anthropometric status at birth also had normal anthropometric status at 22–24 weeks of age (Table 3). Most infants (222/223; 99%) were not receiving any nutritional supplements, such as iron, from birth to 6 months. At 8–10 weeks, 80% (183/228) of mothers were still EBF, which dropped to 60% (136/223) by 6 months.

**Table 2:** Infants’ growth and nutritional status

Characteristic	At birth n = 233	8–10 weeks *n* = 228^a^	22–24 weeks *n* = 223^b^
Weight (grams), mean (SD)	3098 (320)	5120 (703)	7100 (916)
Length (cms), median (IQR)	48 (47–49)	57 (56–58)	65 (63–67)
Weight for age^c^ N (%)			
Normal	232 (99)	211(93)	209 (94)
Under nutrition	0 (0)	17(7)	14 (6)
Underweight (MAM)	0 (0)	12 (5)	13 (6)
Severely underweight (SAM)	0 (0)	5 (2)	1 (1)
Length for age^d^, N (%)			
Normal	215 (94)	211 (93)	195 (87)
Under nutrition	15 (7)	17 (8)	28 (12)
Stunted (MAM)	11 (5)	11(5)	21 (9)
Severely stunted (SAM)	4 (2)	6 (3)	7 (3)
Weight for length^e^, N (%)			
Normal	169 (75)	162 (71)	170 (76)
Possible risk of overweight	44(20)	41 (18)	37 (16)
Over nutrition	12 (5)	11 (5)	10(4)
Overweight	12 (5)	8 (4)	7 (3)
Obese	0 (0)	3 (1)	3 (1)
Under nutrition	1 (1)	14 (6)	8 (4)
Wasted (MAM)	0 (0)	11 (5)	6 (3)
Severely wasted (SAM)	1 (1)	3 (1)	2 (1)
Supplementation from birth, N (%)			
No supplementation	na	221 (97)	222 (99)
Supplemented		7 (3)	3 (1)
Yes, iron	na	1 (0)	0 (0)
Yes, multivitamin	na	2 (1)	3 (1)
Yes, not specified	na	4 (2)	0 (0)

Moderate Acute Malnutrition (MAM) defined when WHO anthropometric measurement is 2 standard deviations (SD) below the mean of normal range and Severe Acure Malnutrition (SAM) defined when WHO anthropometric measurement 3 SDs below the mean of normal range

^a^missing for 5 participants

^b^missing for 10 participants, na = not applicable as this question was not asked in the first interview

^c, d, e^Missing values anthropometric measurement at birth (weight for age = 1, length for age = 3, weight for length = 7), at 6 month (weight for length = 3)

**Table 3:** Growth velocity from birth to 22–24 weeks

	Birth	8–10 weeks	22–24 weeks	N (%)
Weight for age	Normal	Normal	Normal	194 (91)
	Normal	< − 2 SD	Normal	11 (5)
	Normal	Normal	< − 2 SD	9 (4)
	Normal	< − 2 SD	< − 2 SD	5 (2)
	Total			219
Length for age	Normal	Normal	Normal	165 (78)
	Normal	< − 2 SD	Normal	7 (3)
	Normal	Normal	< − 2 SD	15 (7)
	Normal	< − 2 SD	< − 2 SD	10 (5)
	< − 2 SD	Normal	Normal	11 (5)
	< − 2 SD	< − 2 SD	Normal	1(1)
	< − 2 SD	Normal	< − 2 SD	3 (1)
	< − 2 SD	< − 2 SD	< − 2 SD	0 (0)
	Total			212
Weight for length	Normal	Normal	Normal	171 (91)
	Normal	< − 2 SD	Normal	10 (5)
	Normal	Normal	< − 2 SD	5 (3)
	Normal	< − 2 SD	< − 2 SD	0 (0)
	< − 2 SD	Normal	Normal	1 (1)
	< − 2 SD	< − 2 SD	Normal	0 (0)
	< − 2 SD	Normal	< − 2 SD	0 (0)
	< − 2 SD	< − 2 SD	< − 2 SD	0 (0)
	Total			187

Among those who breastfed their infants, mothers reported that the strongest support for EBF was reported to be from the mothers themselves 70% (101/223) and 25% (36/223) from their partners (Table 4). The most common reason for discontinuing EBF at 6 months was mothers needing to return to work (18/223; 25%), followed by inadequate breast milk supply (12/223; 16%) and lack of maternal confidence that breast milk was adequate nutrition (12/223; 16%). Most mothers were feeding (breast milk and/or formula) their infants very frequently, with 69% (158/223) feeding their child over 10 times a day by 22–24 weeks of age.

**Table 4:** Breastfeeding status

Feeding status	8–10 weeks *N* = 228 n (%)	24 weeks *N* = 223 n (%)
Number exclusively breastfeeding (EBF), n (%)	183 (80)	136 (60)
Introduced to formula milk, n (%)	36 (16)	33(15)
Introduced to solid food, n (%)	4 (2)	33 (15)
Introduced to both solid food and formula milk, n (%)	1(0)	22 (10)
Primary support provided for EBF^b^		
Mother herself	148 (80)	101 (70)
Mother’s partner	28 (15)	36 (25)
Mother’s parent/in law	3 (2)	0 (0)
Mother’s friends	1 (0)	0 (0)
Other	5 (3)	8 (5)
Reason discontinuing EBF^c^	*N* = 45	*N* = 87
Inadequate breast milk supply	8 (19)	12 (16)
Not confident breast milk will be enough	5 (12)	12 (16)
Mother was sick/had to take medication	2 (5)	2 (3)
Mother had to get back to work	11 (26)	18 (25)
Other reasons^a^	10 (24)	24 (33)
Feeding frequency in a day (EBF and formula)		
Less than 6 times	4 (2)	5 (2)
6–8 times	12 (5)	11 (5)
8–10 times	34 (15)	54 (24)
More than 10 times	177 (78)	153 (69)

^a^Other reasons included baby kept crying after feeding, trial and error with formula and breast milk did not come in straightaway after birth. EBF = infants who only received breast milk in the first 6-month of life without any additional food or formula

^b^Reported by the mother as who gave her the strongest support to continue breastfeeding Captured only for those still exclusively breastfeeding

^c^some participants had more than one answer

### Infants’ health and case management of ARI and diarrhea

At 8–10 weeks of age, 42% (5/12) of infants with reported ARI symptoms and cough in the 2 weeks prior had received cough expectorants (glycerylguaiacolate (GG)) and 25% (3/12) had received an oral bronchodilator (Table 5). Oral anti-histamines were also commonly prescribed, with a quarter of infants with cough (3/12) and one third of infants with ARI symptoms without a cough (5/17) receiving chlorpheniramine maleate (CTM). At 22–24 weeks, more than half of the infants with a cough in the 2 weeks prior had received expectorants and antihistamines (21/39 and 27/39) and 26% (10/39) and 15% (6/39) of these infants had received mucolytics and bronchodilators respectively (Table 5). With regards to an episode of diarrhea within 2 weeks of the 8–10 and 22–24 week assessments, 8% (*n* = 19) and 9% (*n* = 21) of children, respectively, had experienced diarrhea. The median frequency of diarrhea was 5 (IQR 4–7) and 4 (IQR 4–6) stools a day and the median duration of diarrhea was 2 (IQR 2–4) and 3 (IQR 2–4) days at 8–10 weeks and 22–24 weeks respectively. Use of ORS and zinc for treatment of diarrhea was low, with 5% (1/19) and 14% (3/21) of the infants experiencing diarrhea receiving ORS and 21% (4/19) and 10% (2/21) receiving zinc therapy, at 8–10 and 22–24 weeks of age respectively (Table 6). No hospitalization was reported for any episode of diarrhea.

**Table 5:** Common prescriptions for acute respiratory symptoms in participants with an acute respiratory illness within the 2 weeks prior to the follow-up assessment

Medication type	Age 8–10 weeks		Age 22–24 weeks	
	ARI with cough (*N* = 12)	ARI without cough (*N* = 17)	ARI with cough (*N* = 39)	ARI without cough (N = 23)
No medication, n (%)	1 (8)	1 (6)	2 (5)	1 (4)
Cough medication (Glycerylguaiacolate), n (%)	5 (42)	0 (0)	21 (54)	4 (17)
Antihistamine (CTM), n (%)	3 (25)	5 (29)	27 (69)	10 (44)
Mucolytic (Ambroxol), n (%)	0 (0)	1 (6)	10 (26)	4 (17)
Oral Bronchodilator (Salbutamol), n (%)	3 (25)	2 (12)	6 (15)	3 (13)
Antibiotic (Amoxicillin), n (%)	3 (25)	1 (6)	2 (5)	1 (4)

Results are number (percentage)

**Table 6:** Common prescriptions for diarrhea in participants with an episode of diarrhea within the 2 weeks prior to the follow-up assessment

Medication type	Age 8–10 weeks	Age 22–24 weeks
	(*N* = 19)	(*N* = 21)
No rehydration	11 (58)	13 (62)
ORS rehydration at any time	1 (5)	3 (14)
Zinc tablet	4 (21)	2 (10)
ORS rehydration at any time and zinc tablet	3 (16)	1 (5)

Results are number (percentage). ORS = Oral Rehydration Solution; A liquid given orally to prevent or correct dehydration due to diarrhea disease

## Discussion

This is one of the first studies to describe the nutritional status, feeding practice and health status particularly ARI and diarrhea case management, in the first 6 months of life in Indonesia. The study was conducted alongside a rotavirus vaccine clinical trial at three separate time points after birth and has provided valuable insights into breastfeeding practices and the management of both ARI and diarrhea in two regions of Indonesia.

At the both points after birth, 5–6% of our study participants were underweight, 1–2% were severely underweight; 3–5% were wasted and 1% were severely wasted. We also found that at all three time points stunting occurred in 5–9% of study participants, with 2–3% being severely stunted. This is substantially lower than national finding in 2013 where the proportion of underweight, wasted and stunted infants in Indonesia was 19.6, 37.2 and 12.1% respectively [6]. As expected, the overall nutritional status of infants within our study was higher than the general population of infants in Indonesia since they had higher engagement with the health system through the clinical trial and improved access to early detection of nutritional problems compared with the general population. [28]. Nevertheless, it is clear that nutritional problems are still an issue in Indonesia. To address the national problems in under nutrition, the Indonesian government has engaged with the Scaling Up Nutrition (SUN) movement, a program that aims to provide country specific nutritional intervention for pregnant women and children under 5 years (i.e Vitamin A supplementation) and support breastfeeding, to improve the management of under nutrition [29]. In the 3 years since introduction of the SUN movement in 2011, the prevalence of stunted children under 5 years had decreased from 39.2 to 36.4%, with a target of 26.3% by 2025 [30].

In addition to the reported under nutrition in this study, we also identified 5% of infants who were overweight or obese. Moreover, a fifth of the total infants participating in this study were at possible risk of overweight and might need further nutritional evaluation. Improvement in the economic conditions in developing countries may increase the prevalence of overweight and obesity that co-exists with under nutrition through factors such as life styles changes (such as reducing physical activities), urbanisation and aggressive nutrition interventions for undernourished children [31]. In 2014 the Indonesian Pediatric society published a guideline for the management and prevention of childhood obesity. For children aged 0–12 months, exclusive breast-feeding is recommended up to 6 months with continuation of breastfeeding up to 12 months. Other recommendations include the introduction of a wide variety of foods, avoidance of sweetened beverages and snacks and no televisions in bedrooms [32].

In our study, 60% of women were still EBF their infant at 6 months. This was higher than the recent WHO report of 42% EBF coverage in the first 6 month of age in Indonesia in 2014 [33]. In the same year the Indonesian ministry of health reported that the EBF rate was 54% nationwide. The current national Indonesian EBF rate of 46.3% [34] is above the global average rate of EBF (37% across 75 countries), [11] although it is still below the Indonesian government’s target of >75% [35]. The higher EBF rate in our study may be due to the families’ engagement in the vaccine clinical trial, providing a higher exposure to health care providers and to the national program that encouraged EBF. In our study, majority of mothers reported themselves as the primary support for EBF at 6 months, and 25% of mother reported that the strongest support for EBF was from their partners. Other studies have reported that mothers who face difficulties maintaining EBF are more likely to have limited partner support [36]. To reach the targeted level of EBF in Indonesia, increasing support for mothers practicing EBF in the first 6 months, including partner education, and appropriate complementary feeding support up to 24 months, is needed. Most of the infants were reported to be breastfed for more than 10 times per day as recommended by American Academy of Pediatrics [37].

The prescription of cough medication for ARIs in the first 6 months, including expectorants (Glyceryl Guaiacolate or Guaifenesin), antihistamines (Chlorpeniramin maleat) and mucolytics (ambroxol), was very common in our study despite it not being recommended by the Indonesian Pediatric society and the WHO. Expectorants, mucolytic agents in cough medication, antihistamines and oral bronchodilators are not recommended medication for the management of URTI in children. In addition, a systematic review reported that most cough medications in children are not effective [38, 39] and there have been a number of reports of toxicity and deaths related to cough medications in young infants, [40, 41] mostly due to Chlorpheniramin maleat (CTM) and ambroxol. In the current study, CTM is the second most commonly prescribed cough medication in infants, especially in primary health care settings. Studies reported that CTM can depress the central nervous system or cause dysrhythmias [41, 42]. Among 10 cold and cough medication-related deaths in infants <6 months of age in a US study, four had toxic levels of CTM and Ambroxol in their blood [36]. This was possibly because the dosage of cough and cold medication deemed to be safe in infants had been extrapolated from adult data with limited safety data available for use in young infants [40]. Education of mothers and health workers regarding the risk and health benefits for giving the abovementioned therapies in infants is required.

Bronchodilator use in infants under 6 months was also very common in our study for the treatment of cough. WHO guidelines recommend that bronchodilators in ARI are given by inhalation and not by oral therapy [21] and are only recommended when children present with wheeze with unclear cause or wheeze with the presence of fast breathing or chest indrawing, and not for mild cough. There is little evidence of a beneficial effect from giving oral bronchodilators for the treatment of cough alone [21]. A randomised controlled trial in India comparing the use of oral salbutamol for symptomatic relief in acute cough with placebo concluded non-superiority of oral salbutamol [43] and there was also little evidence, that oral salbutamol affected the mean duration of fever, cough, and noisy breathing or the time to the resolution to normal activities compared with placebo. Of most concern, 5 out of 140 Indian infants in the bronchodilator group were reported to have adverse effects including tremors [43, 44]. Salbutamol use in neonates has also been associated with supraventricular tachycardia [45]. Considering there appears to be limited or no benefit from giving oral salbutamol for the treatment of mild ARI in infants, and there are substantial safety concerns, administration of oral salbutamol is not recommended for infants and further education of health care providers in Indonesia is needed.

The use of ORS and zinc supplementation to treat diarrhea was low in our study. Currently, WHO recommends rehydration with ORS and zinc supplementation daily for 10–14 days for the treatment of acute diarrhea episodes with no or some dehydration [21]. However, most diarrhea episodes in our study were mild, with diarrhea occurring on average 4–5 times a day and lasting for 3 days during an average episode, and none required hospitalisation. Yet, the findings from our study are consistent with the low use of ORS for rehydration and zinc supplementation for treatment of diarrhea that has been identified nationally for the management of acute diarrhea, where it was reported that 33 and 17% of children under five with acute diarrhea received ORS and zinc respectively [6]. Low implementation of ORS might be due to home solutions being given over ORS (due to the ease of availability and for palatability reasons) or due to low adherence to WHO guidelines in community settings, once again raising the need for further education of healthcare workers.

There were several limitations to the current study. The general health of infants in this study would be higher than the general population as all the participants were enrolled in a clinical trial, were regularly monitored and had good engagement with the health system. However, we still found evidence of wasting, severe stunting and obesity in up to 9% of infants. In this study, we did not collect information for all episodes of ARI and diarrhea over the 6 months but rather chose to focus on case management of ARI and diarrhea in episodes that occurred within 2 weeks prior to the follow-up assessments. Given this was not our aim, we were unable to provide an estimate of the incidence of ARI and diarrhea episodes, nor report the treatment received for all episodes of ARI or diarrhea occurring within the first 6 months of life. In addition, we did not collect severity of diarrhea within this study, which may have been lower as participants were included in a rotavirus vaccine clinical trial, although both vaccine and placebo participants were included. Diarrhea severity would also have impacted on the use of ORS and zinc, but regardless use was reported to be very low with poor adherence to guidelines for diarrhea case management. There was also the potential for recall bias with ARI and diarrhea episode management, with episodes retrospectively reported by the mother. Further clarification was sought from medical reports held at the PHCs on treatment but completeness of the diagnostic and management information varied.

## Conclusion

In a prospective cohort study of the nutritional status and management of acute respiratory illness and diarrhea in infants enrolled in a vaccine clinical trial in Indonesia, EBF rates at 6 months were below the Indonesian national target. Adherence to recommended WHO guidelines for case management of ARI and diarrhea in the first 6 months of life was poor, with a high use of cough medicines and oral bronchodilators. Our findings highlight the need for ongoing education of community health providers in the support of EBF and optimal case management for childhood ARI and diarrhea in Indonesia.

## Abbreviations

ARI: Acute respiratory illnesses
CRF: Case report forms
CTM: Chlorpheniramine maleate
EBF: Exclusively breast feeding
GG: Glycerylguaiacolate
MAM: Moderate acute malnutrition
MDG: Millennium development goal
ORS: Oral rehydration solutions
PHCs: Primary health care clinics
SAM: Severe acute malnutrition
UNICEF: The United Nations children’s emergency fund
URTI: Upper respiratory tract infections
WHO: World Health Organization
